# The effect of Baduanjin exercise on health-related physical fitness of college students: study protocol for a randomized controlled trial

**DOI:** 10.1186/s13063-019-3672-1

**Published:** 2019-09-18

**Authors:** Fang Zhao, Shanshan Sun, Jian Xiong, Guohua Zheng

**Affiliations:** 1 College of Nursing and Health Management, Shanghai University of Medicine and Health Sciences, Shanghai, 201318 China; 2 Graduate School, Shanghai University of Traditional Chinese Medicine, Shanghai, 201303 China

**Keywords:** College students, Baduanjin exercise, Physical fitness

## Abstract

**Background:**

Low health-related physical fitness in college students is a risk factor for future development of cardiovascular diseases at later stages in life, but appropriate exercise is one of the main determinant factors of health-related physical fitness. Previous studies have showed that Baduanjin exercise is beneficial in improving sleep quality, mental health, body flexibility, and body physique. However, the evidence is unclear whether Baduanjin exercise can be recommended as an effective exercise to promote health-related physical fitness of college students.

**Methods/design:**

One hundred twenty college students will be recruited and randomly allocated to either the Baduanjin exercise or the control group at a ratio of 1:1. The students in the Baduanjin exercise group will receive a 12-week supervised Baduanjin exercise training intervention with a frequency of 1 h each day and 4 days per week, while those in the control group will not receive any specific exercise intervention and will be informed to maintain their original lifestyle for 12 weeks. The primary outcome of health-related physical fitness involving measurements of body flexibility, muscular strength, cardiopulmonary fitness, and body endurance will be measured at baseline and after the 12-week intervention period. Mixed linear models will be used to analyze the effect of the Baduanjin exercise intervention on the health-related physical fitness of college students.

**Discussion:**

This is the first trial to evaluate the effects of Baduanjin exercise on health-related physical fitness in college students. If the results are as expected, they will provide evidence of Baduanjin exercise in promoting health-related physical fitness in young adults.

**Trial registration:**

Chinese Clinical Trial Registry, ChiCTR-IOR-17013011. Registered on 17 November 2017.

**Electronic supplementary material:**

The online version of this article (10.1186/s13063-019-3672-1) contains supplementary material, which is available to authorized users.

## Background

Health-related physical fitness is defined as the capability of an individual to carry out his everyday activities without excessive fatigue and with enough spare energy to enjoy free time and to solve unusual situations [1, 2]. Health-related physical fitness is also regarded as an integrative measure of most body functions, such as musculoskeletal, cardiorespiratory, hematocirculatory, endocrine-metabolic, and psychoneurological functions [3, 4].

With China’s rapid economic development and transitions in lifestyle, the health-related physical fitness of its adolescent and students has undergone a significant decline. For example, the rates of overweight and obesity in 2010 were estimated to be 9.9% and 5.1%, respectively, in school-aged adolescents, and neared those of developed countries [5]. Furthermore, the measure of physical endurance of students was not optimistic, and the failure rate of endurance running was up to 23.7% in male students and 19.6% in female students [6]. According to reports from National Student Physical Fitness Research, the health-related physical fitness of students in China gradually declined from 1985 to 2005 [7]. This tendency was curbed in 2010, but a significant improvement was still not achieved [8, 9].

College students are in a critical period of growth and development that connects high school with adulthood [10]. They will complete the transition to autonomy by independently making decisions and developing behavior patterns, many of which will continue throughout their lives [11]. Due to higher study pressures and the pervasive presence of the Internet and smartphones, college students are prone to developing unhealthy behaviors and habits, such as physical inactivity, poor diet, and alcohol misuse [12, 13]. Low health-related physical fitness in young adults is a risk factor for the future development of cardiovascular disease, which has globally increased in the last five decades. This represents a public health issue in the young population about which we should be concerned [14, 15].

Health-related physical fitness is partially genetically determined, but it can be strongly influenced by environmental factors [16, 17]. Appropriate exercise is one of the main determinant environmental factors [13, 18]. Regular exercise can not only increase the level of physical activity and correct the deviation of health-related behavior, it can also aid in the development of a healthful lifestyle [19, 20]. Qigong is the general name given to many traditional Chinese mind–body exercises [21]. It is a holistic system of coordinated body posture and movement, breathing, and meditation used for health, spirituality, and martial arts training, and it has been practiced for health promotion in China for thousands of years [22–24]. Baduanjin exercise, one of the most common forms of Qigong, consists of eight separate, delicate, and smooth exercise movements; each section brings certain function-enhancing benefits to different physical parts of the body or particular organs [25]. Through concepts and respiratory adjustments designed to achieve self-psychosomatic regulation, Baduanjin exercises emphasize the meaning of “Qi” and “form,” resulting in a comprehensive exercise to achieve physical and mental harmony [25]. Studies have shown that Baduanjin training is helpful for older people with physical and mental disorders such as anxiety, hyperlipidemia, spinal problems, osteoarthrosis, and type 2 diabetes [26–28]. Several studies also indicated that Baduanjin exercise has a potential benefit in improving physical function in young adults [29–31]. Our previous study indicated that a 12-week regimen of regular Baduanjin training could improve the lower limb proprioception and flexibility of college students [32, 33], but other parameters of health-related physical fitness such as muscular strength, endurance, cardiovascular fitness, and body composition were not observed. Therefore, the evidence is unclear whether Baduanjin exercise can be recommended as an effective exercise to improve the health-related physical fitness of young adults. The purpose of this trial is to evaluate the effects of Baduanjin exercise on health-related physical fitness of college students by a randomized controlled trial with a rigorous design.

## Methods/design

### Study aim

The aim of this trial is to evaluate the effectiveness of a 12-week Baduanjin exercise intervention on the health-related physical fitness of college students.

### Study design

**Fig. 1:**
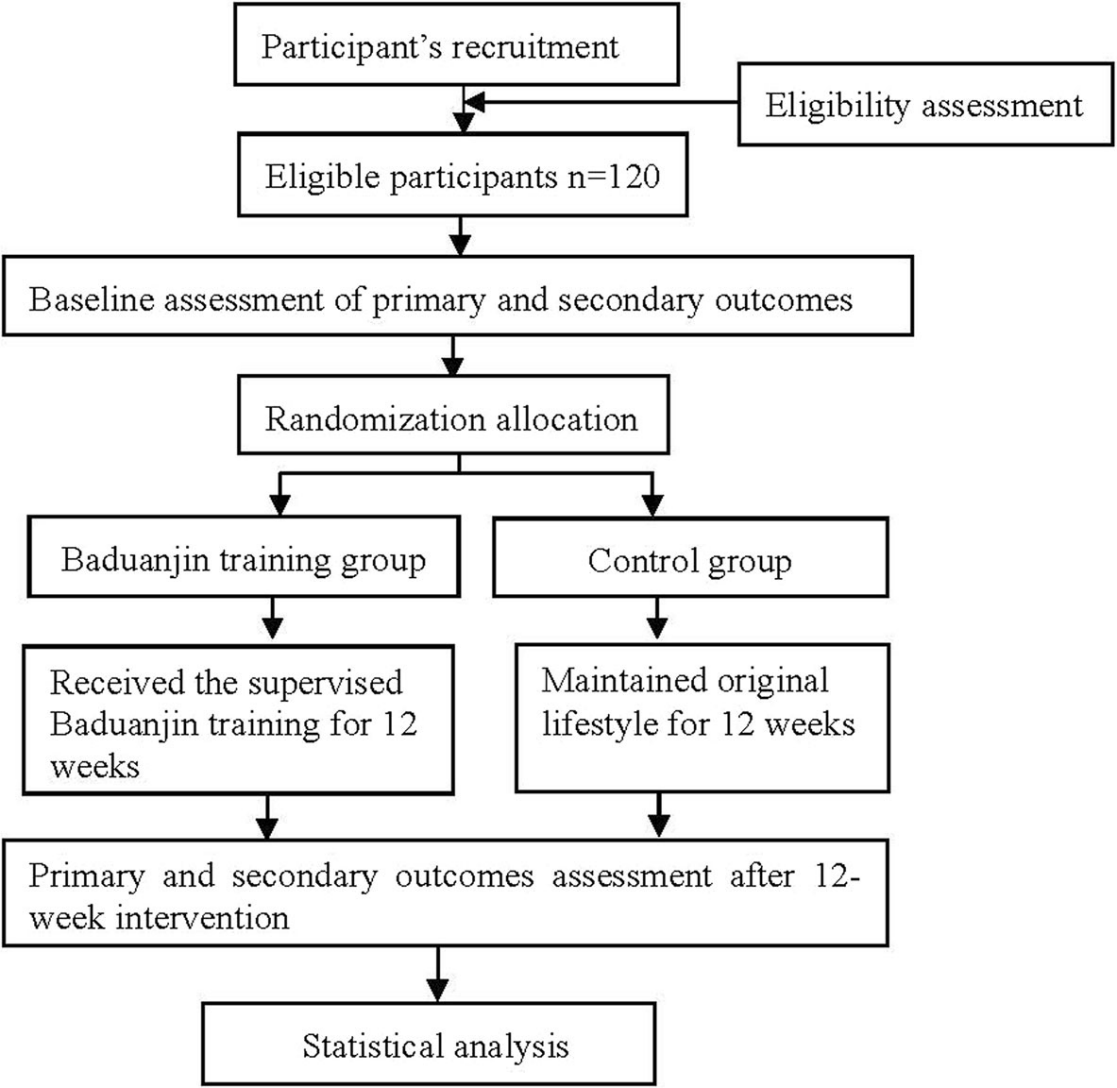
Proposed participant flow (Consolidated Standards of Reporting Trials [CONSORT] diagram)

This study is a randomized, parallel-group controlled trial to evaluate the effectiveness of a Baduanjin exercise intervention on the physical fitness of college students. A total of 120 eligible college students from the Shanghai University of Medicine and Health Sciences (SUMHS) will be recruited and randomly allocated to either the Baduanjin exercise group or the control group at a ratio of 1:1. Participants in the Baduanjin exercise group will receive a 12-week supervised Baduanjin exercise training intervention with a frequency of 1 h each day and 4 days per week, while those in the control group will not receive any specific exercise intervention and will be informed to maintain their original lifestyle for 12 weeks. Primary and secondary outcomes will be measured at baseline and after the 12-week intervention period. The participant flow for this trial is presented in Fig. 1. The present protocol follows the Standard Protocol Items: Recommendations for Interventional Trials (SPIRIT) guidelines and fulfills the SPIRIT checklist (see Additional file 1).

### Sample size calculation

The primary outcome of this trial involves global health-related physical fitness ability changes which are assessed using composite points of health-related physical fitness. According to study data [34], the mean and standard deviation of a composite point in the health-related physical fitness of college students were measured as 55 points and 12.5 points, respectively. A sample size of 100 will have 80% power to detect a difference in a composite score of health-related physical fitness of 5.0% and an α of 0.05 for a two-sided significance level. Assuming a maximum loss to follow-up of 20%, a total of 120 subjects was necessary.

### Setting and recruitment

**Fig. 2:**
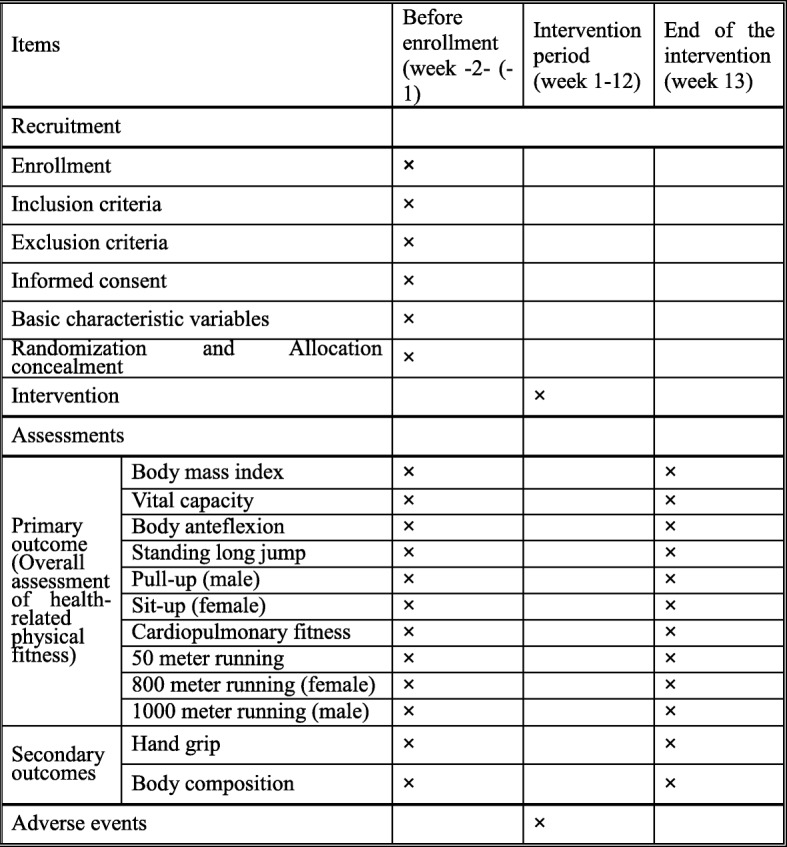
SPIRIT figure showing schedule of interventions and assessments

This study will be performed at SUMHS in China. Data collection and exercise training will occur at the campus of this university. Recruitment of eligible participants will be conducted by two research assistants independently and will be achieved with the use of campus radio, flyers, and WeChat. Figure 2 shows the SPIRIT schedule of assessments and interventions. Formal recruitment for the study began in May 2018.

### Participant eligibility

The eligible participants should fulfill the study inclusion criteria and not comply with the exclusion criteria. For inclusion, the participant should be aged 16 to 25 years, be able to provide a signed informed consent form, and be a full-time student at first or second grade. Criteria for exclusion from the study are as follows: being or having been engaged in a long-term regular practice of Baduanjin; being a member of the Martial Arts Association, Dance Association, Aerobics Association, Sanda Association, or Taekwondo Association; or having had severe cardiovascular diseases, musculoskeletal system diseases, or other sports contraindications.

### Randomization, allocation, and blinding

The random allocation sequence will be produced by an independent statistician via the PLAN sentences of the statistical software SAS 9.1. The random allocation sequence will be managed by an assigned project manager who will not take part in the participants’ recruitment, and it will be concealed from those research assistants who will be responsible for recruitment or outcome assessment. After signing the informed consent and undergoing baseline assessment of primary and secondary outcome measures, the eligible participants will receive their information of allocation result via WeChat. They will be allocated randomly into either the Baduanjin exercise group or the control group.

Although both the participants and exercise coaches cannot be blinded in this trial, we will blind the outcome assessors and statistics analyzers by the following procedures. Participants’ outcomes will be measured by two special investigators who are not involved in the allocation and exercise intervention. Before the outcome measurement starts, participants, exercise coaches, and the related research assistants will be told not to reveal any information about the allocation. The statistical analysis will be performed by a statistician who does not participate in the implementation of the trial, and the allocation information of the groups will be concealed by a blind code. The blind code will be disclosed when the statistical analysis is completed.

### Intervention

#### Baduanjin exercise group

**Fig. 3:**
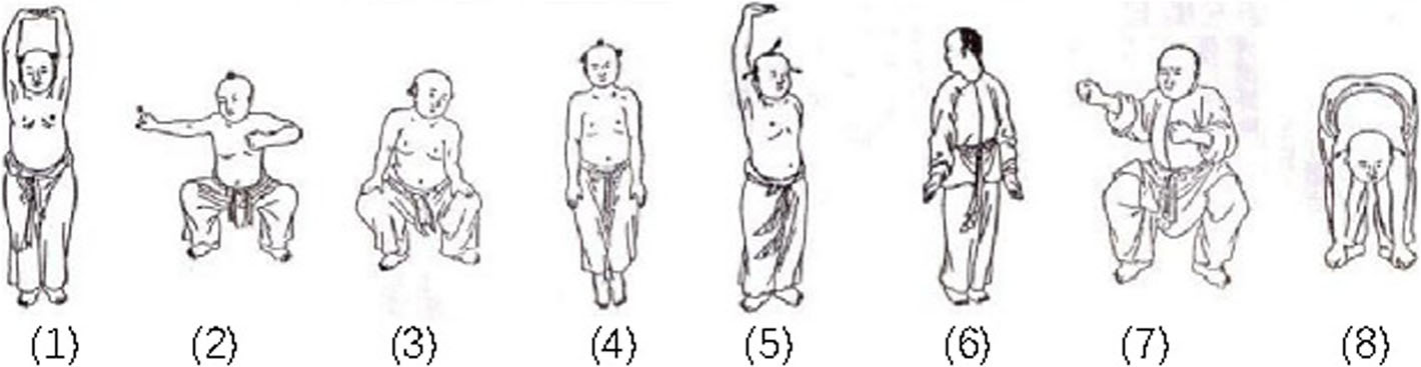
The Baduanjin exercise postures

Participants in the Baduanjin exercise group will be assembled at the gymnasiums of the university and will receive Baduanjin exercise training at a frequency of 4 days per week, 1 h per day, for 12 weeks. The training scheme originated from *Health Qigong - Baduanjin,* published by the General Administration of Sport of China [35]. The whole set of Baduanjin exercises consists of eight postures (Fig. 3). Two qualified coaches will be employed to teach the participants the correct Baduanjin postures and supervise them during the entire intervention period.

#### Control group

The participants in the control group will not receive any specific exercise training during the 12-week intervention period. They will be instructed to maintain their original lifestyle.

All participants in both the Baduanjin exercise group and the control group will be required to record their daily physical activity by using a step counter in their mobile phone and to record exercise information during the 12-week intervention period.

#### Outcome assessment

Outcome measurement of participants will be performed at the campus gymnasium by physical education teachers who do not participate in the implementation of this trial. Primary and secondary outcomes will be assessed at baseline and after the 12-week intervention period.

#### Primary outcome

According to China’s National Student Physical Health Standard (2014 Revision) [36], the health-related physical fitness measurements consist of body mass index (BMI), vital capacity (VC), 50-m running, body anteflexion in sitting position (BAISP), standing long jump (SLJ), pull-ups per minute (male)/sit-ups per minute (female), and 1000-m running (male)/800-m running (female). Overall assessment of health-related physical fitness is evaluated using a composite point which is calculated as the summation of the points of each measurement multiplied by the participant’s corresponding weight. A composite point of health-related physical fitness = 15%BMI points + 15%VC points + 20% 50 m running points + 10% BAISP points + 10% SLJ points + 10% pull-up (male)/sit-up (female) points + 20% 1000 m (male)/800 m (female) running points. The scoring standard and weight of each measurement are listed in Tables 1 and 2.

- Body mass index will be assessed using the formula BMI = body mass (kg)/height (m)^2^. Body mass and height will be measured using the Height and Weight measuring instrument produced by Zhongtitongfang Co., Ltd., Beijing (product type ZTTF-CSTF-ST).
- Vital capacity, the maximum amount of air a person can expel from the lungs after a maximum inhalation, will be measured using the vital capacity tester produced by Zhongtitongfang Co., Ltd., Beijing (product type CSTF-FH-5000).
- Body anteflexion in sitting position is applied to evaluate the body flexibility of the lower back and hamstring muscles and will be measured by the “sit and reach” test using a “sit and reach” tester produced by Zhongtitongfang Co. (product type CSTF-TQ-5000). For the test procedure, the subject sits on the floor with legs stretched out straight ahead and the soles placed flat against the box; then the hands with the palms facing downward reach forward along the measuring line as far as possible. The point is recorded to the nearest centimeter as the distance reached by the hand.
- Standing long jump is a common test to assess the explosive power of the student’s legs and will be measured using the longitudinal jump tester produced by Zhongtitongfang Co. (product type CSTF-ZL-5000). The student stands behind a line marked on the ground with feet slightly apart, then attempts to jump as far as possible. The scoring is the distance jumped from take-off line to the nearest point of contact on the landing.
- Pull-ups are a common way to measure the upper body strength and will be measured using the chin-up tester produced by Zhongtitongfang Co. (product type CSTF-YT-5000). The subject grasps the overhead pull-up bar, then pulls the body up until the chin extends the bar. The point is the number of pull-ups per minute.
- Sit-ups measure the strength of the abdominal muscles and will be measured using the sit-ups tester produced by Zhongtitongfang Co. (product type CSTF-YW-5000). The subject lays down with the knees bent and the feet placed flat on the ground and the arms crossed over the chest; she then slowly sits up until the body is vertical, then slowly lays back down. The point is the number of sit-ups per minute.
- Cardiopulmonary fitness will be assessed using the step test, which is measured with the step tester produced by Zhongtitongfang Co. (product type CSTF-TZ-5000). The subject completes 30 up and down steps per minute for 3 min in accord with the rhythm, then measures the pulse at 1–1.5 min, 2–2.5 min, and 3–3.5 min after stopping the test. The index of step test = (duration of step moving (in seconds) × 100)/(2 × (sum total of three measured pulses)).
- The 50-, 800-, and 1000-m running test evaluates the capability of rapid running and reaction as well as aerobic fitness. It will be measured by the physical education teachers using a stopwatch at the campus recreation ground.

**Table 1:** Scoring standard and weight of body mass index for health-related physical fitness of college students

BMI	100 (points)	80 (points)	60 (points)	Weight
Male	17.9–23.9 (kg/m^2^)	≤ 17.8 or 24.0–27.9 (kg/m^2^)	≥ 28.0 (kg/m^2^)	15%
Female	17.2–23.9 (kg/m^2^)	≤ 17.1 or 24.0–27.9 (kg/m^2^)	≥ 28.0 (kg/m^2^)	15%

**Table 2:** Scoring standard and weight of each measurement for health-related physical fitness of college students

Points	Male						Female					
	VC (ml) W:15%	50 m (s) W:20%	BAISP (cm) W:10%	SLJ (cm) W:10%	Pull-ups (times/min) W:10%	1000 m (min:s) W:20%	VC (ml) W:15%	50 m (s) W:20%	BAISP (cm) W:10%	SLJ (cm) W:10%	Sit-ups (times/min) W:10%	800 m (min:s) W:20%
100	5040	6.7	24.9	273	19	3′17”	3400	7.5	25.8	207	56	3′18”
95	4920	6.8	23.1	268	18	3′22”	3350	7.6	24.0	201	54	3′24”
90	4800	6.9	21.3	263	17	3′27”	3300	7.7	22.2	195	52	3′30”
85	4550	7.0	19.5	256	16	3′34”	3150	8.0	20.6	188	49	3′37”
80	4300	7.1	17.7	248	15	3′42”	3000	8.3	19.0	181	46	3′44”
78	4180	7.3	16.3	244		3′47”	2900	8.5	17.7	178	44	3′49”
76	4060	7.5	14.9	240	14	3′52”	2800	8.7	16.4	175	42	3′54”
74	3940	7.7	13.5	236		3′57”	2700	8.9	15.1	172	40	3′59”
72	3820	7.9	12.1	232	13	4′02”	2600	9.1	13.8	169	38	4′04”
70	3700	8.1	10.7	228		4′07”	2500	9.3	12.5	166	36	4′09”
68	3580	8.3	9.3	224	12	4′12”	2400	9.5	11.2	163	34	4′14”
66	3460	8.5	7.9	220		4′17”	2300	9.7	9.9	160	32	4′19”
64	3340	8.7	6.5	216	11	4′22”	2200	9.9	8.6	157	30	4′24”
62	3220	8.9	5.1	212		4′27”	2100	10.1	7.3	154	28	4′29”
60	3100	9.1	3.7	208	10	4′32”	2000	10.3	6.0	151	26	4′34”
50	2940	9.3	2.7	203	9	4′52”	1960	10.5	5.2	146	24	4′44”
40	2780	9.5	1.7	198	8	5′12”	1920	10.7	4.4	141	22	4′54”
30	2620	9.7	0.7	193	7	5′32”	1880	10.9	3.6	136	20	5′04”
20	2460	9.9	−0.3	188	6	5′52”	1840	11.1	2.8	131	18	5′14”
10	2300	10.1	−1.3	183	5	6′12”	1800	11.3	2.0	126	16	5′24”

Abbreviations: *W* weight, *VC* vital capacity, *BAISP* body anteflexion in sitting position, *SLJ* standing long jump

#### Secondary outcomes

Handgrip strength, which is used to determine the maximum isometric strength of the hand and forearm muscles, will be measured using the handgrip strength dynamometer produced by Zhongtitongfang Co. (product type CSTF-WL-5000). The subject holds the dynamometer in the hand with the arm at a right angle and the elbow by the side of the body, then squeezes the dynamometer with maximum isometric effort. The best result from several tests for each hand will be recorded.

Body composition includes fat mass, body fat (percentage), fat-free mass, and lean body mass. These measurements will be taken by bioelectrical impedance analysis using an Inbody 720 (Biospace Co.), which emits a low-intensity electrical current that runs through the person’s body to measure resistance, reactance, and phase angle.

#### Safety measurements

Any unexpected adverse events (AEs) that occur during the 12-week intervention period will be reported to the research assistants, and the causality of Baduanjin exercise will be assessed. If serious AEs occur, the research assistants will report them to the project manager and ethics committee immediately; they will make a decision on whether the participant needs to withdraw from the study.

#### Data collection

The demographic characteristics will be collected by the research assistants at the recruitment. The primary and secondary outcome data will be collected by the outcome assessors at baseline and at the end of the intervention. All data collection in this study will be performed using standardized procedures, and the outcome assessors will receive standard training on how to measure all outcomes to ensure equal testing conditions for all participants.

To promote participant retention and the provision of complete outcome data from all participants, a total incentive of up to 200 RMB per individual as compensation for their time participating in the study will be provided to all participants. The incentive will be delivered using the way of WeChat draw via WeChat after completion of the final assessment following the 12-week intervention.

#### Data management

The outcome assessors will be responsible for filling in the printed case report form (p-CRF) when they measure the primary and secondary outcomes at each timeline. The research assistants will be responsible for checking the integrity of the completed p-CRF and for timely inputting of the collected data into the EpiData Manager, a free data management software. The project manager will be responsible for initial data cleaning, identifying, and coding and for converting the data into the proper format for analysis.

### Statistical analysis

Analysis of data in this trial will be performed by a statistician who is not involved in the trial. In descriptive analysis of the sample, continuous variables will be expressed by using the mean and standard deviation for normal distributions, and the median and interquartile range for non-normal distributions. Normality will be tested using the Kolmogorov–Smirnov test. Appropriate transformations will be applied in cases of non-normal distribution. Categorical variables will be expressed as proportions with their standard error.

Baseline characteristics between groups will be compared using the *t* test or Mann–Whitney test for continuous variables and Pearson’s χ-squared or Fisher’s exact test for categorical variables. If incomparability appears, the inequality factors will be treated as confounding variables in the final efficacy analysis.

For comparison of primary and secondary outcomes between groups, a *t* test or non-parametric tests will be used for continuous data, and Pearson’s χ-squared or Fisher’s exact test for categorical data at baseline and after intervention; the between-group differences (treatment effects) will be analyzed by using mixed linear models with restricted maximum likelihood using the group-by-time interaction terms. Analysis of the outcomes will be performed on the basis of the intention-to-treat (ITT) population. All participants who have been randomized will be included in the final analysis irrespective of compliance or withdrawal from the program. Full information maximum likelihood estimation will be employed for missing data.

AEs will be listed and analyzed using a χ-squared test or Fisher’s exact test. Severe AEs will be listed and described in detail.

All data will be analyzed with SPSS 21.0 (IBM, Chicago, IL, USA) software packages. Statistical significance is defined as a two-sided *P* value of < 0.05.

### Ethics

The conduct of the study will conform to the principles of the Declaration of Helsinki and relevant ethical guidelines covering informed consent, confidentiality, and data storage. Ethical approval has been obtained from the Ethics Committee of Shanghai University of Medicine and Health Sciences (approval number 2017ZGH). All participants will be fully informed about the trial and will sign the informed consent form prior to participation.

### Monitoring

Due to the low-risk nature of the aerobic exercise intervention, we do not anticipate any potential harms. Therefore, there will be no Data Monitoring Committee, interim analyses, or stopping rules.

### Dissemination

The study protocol has been registered, and is available on the Chinese Trial Registry website (registered in ChiCTR.org with the identifier ChiCTR-IOR-17013011). The results will be disseminated to all participants, researchers, healthcare providers, and sponsors through study summary documents, courses, presentations, and the Internet. This study will also be published in scientific journals and be presented at conferences to target a wide range of groups.

## Discussion

Compared with conventional exercise styles (e.g., resistance exercise, muscular endurance exercise, and aerobic exercise), Baduanjin typically involves a mind–body integration practice to balance the Yin and Yang in the body and promote blood circulation and Qi for maximizing both physical and mental well-being [37]. Previous studies have shown that regular Baduanjin exercise is beneficial in improving psychological and physiological outcomes among elderly adults and various clinical populations (e.g., Parkinson’s disease, chronic neck pain, chronic fatigue syndrome-like illness, psychological illness) [38–40]. As a typical mind–body exercise with characteristics of traditional Chinese medicine, Baduanjin may be considered to be an effective exercise for a wide range of populations (e.g., a healthy population or patients with chronic diseases; young, middle-aged, or elderly adults) in promoting health. In recent years, the effect of Baduanjin in health promotion has gradually been accepted by young adults, especially college students in China. An increasing number of studies have found that regular practice of Baduanjin can modulate sub-health [41], improve the body physique and mental health [42, 43], promote sleep quality [44], and motivate Qi circulation [45] in college students. In our previous trial, we found that 12 weeks of regular Baduanjin training could improve the lower limb proprioception and flexibility in college students [33]. In this trial, we will observe the effect of 12 weeks of Baduanjin training on the health-related physical fitness of college students. A rigorous study design with randomized allocation and blinding to assessors and statistical analyzers will be applied to reduce bias. It is expected that this trial will create reliable results.

There are some potential limitations to this study. First, it is difficult to monitor the additional physical activity of participants during the study duration. Although all participants will be required to record their daily physical activity or exercise information by using a step counter in their phone, this is not accurate enough to measure their daily activity intensity. Second, due to unexpected accidents such as training time conflicts with other commitments and bad weather, individual adherence to the Baduanjin exercise regimen in the Baduanjin exercise group may be another issue which can impact the study results. Furthermore, blinding of participants is impossible in this trial, because of the nature of the exercise intervention. However, we will make every effort to ensure that outcome assessors, data managers, and statisticians are unaware of the treatment allocations.

Altogether, the results of this trial may provide evidence of whether regular Baduanjin training can improve the health-related physical fitness of college students in China.

### Trial status

This trial is ongoing. participant recruitment began on May 1, 2018, and completed on April 30, 2019. The trial procedures are expected to be completed by the end of October 2019.

## Additional file

Additional file 1 SPIRIT 2013 checklist: recommended items to address in a clinical trial protocol and related documents. (DOC 91 kb)

## Data Availability

Data for the study can be made available upon request. Interested researchers should contact Dr. Zheng at zhenggh@sumhs.edu.cn.

## Abbreviations

AEs: Adverse events
BAISP: Body anteflexion in sitting position
BMI: Body mass index
CONSORT: Consolidated Standards of Reporting Trials Consolidated Standards of Reporting Trials
CRF: Case report form
ITT: Intention-to-treat
SLJ: Standing long jump
SPIRIT: Standard Protocol Items: Recommendations for Interventional Trials
VC: Vital capacity
